# Expression of p16 and Ki67 in laryngeal squamous cell carcinoma and their clinical significance

**DOI:** 10.3389/fonc.2024.1430830

**Published:** 2024-09-23

**Authors:** Xue-Ying Liu, Shangguan Hanjing, Bing-huang Zhang, Xian-Yang Luo

**Affiliations:** Department of Otolaryngology Head and Neck Surgery, The First Affiliated Hospital of Xiamen University, School of Medicine, Xiamen University, Xiamen, China

**Keywords:** clinical significance, Ki67, laryngeal squamous cell carcinoma, p16, laryngeal squamous cell

## Abstract

**Objective:**

The objective of this study is to assess the prognostic value of Ki67 and p16 proteins in laryngeal cancer.

**Materials and methods:**

This retrospective cohort analysis comprised 260 patients diagnosed with laryngeal cancer. Immunohistochemistry (IHC) was employed to assess the expression levels of p16 and Ki67, and their correlation with the survival time of laryngeal cancer patients was analyzed.

**Results:**

The Ki67 index level exhibited a significant association with the prognosis of laryngeal cancer. Patients with higher Ki67 index levels demonstrated shorter survival times, more severe pathological classification, and higher tumor stages (P < 0.05). Conversely, no significant differences in prognostic characteristics of laryngeal cancer were observed in the p16 (-/+) population (*P* > 0.05). The median survival times for patients with Ki67 index levels of 0-35%, 36-70%, and 70-100% were 3.54 years, 2.10 years, and 1.92 years, respectively. After adjusting for age, smoking, alcohol consumption, pathological classification, surgical intervention, recurrence, and metastasis, the risk of death for patients with Ki67 index levels of 70-100% was 2.0504 times higher than that of patients with Ki67 index levels of 0-35% (95% CI: 1.2997-3.2345, *P* = 0.0020).

**Conclusion:**

The Ki67 index level is strongly associated with survival time and the risk of death in laryngeal cancer, making it a valuable prognostic indicator. However, the prognostic value of p16 levels in laryngeal cancer is limited. These findings provide important insights for prognosis evaluation and treatment decision-making in patients with laryngeal cancer.

## 1 Introduction

Laryngeal cancer is a prevalent malignancy that commonly affects the head and neck region, albeit accounting for a relatively small proportion (approximately 1% to 3%) of all malignant tumors in the body. However, it holds a significant incidence among tumors in the ear, nose, and throat, representing approximately 50% of cases, with squamous cell carcinoma being the predominant histological type. Laryngeal cancer can be categorized into supraglottic, glottic, and subglottic types, with the glottic type being the most prevalent, encompassing approximately 60% of cases (1). In recent years, there has been an upward trend in the global incidence and mortality rates associated with laryngeal cancer (2, 3). According to the latest global cancer data in 2020, the worldwide death toll from laryngeal cancer is estimated to be approximately 99,840, indicating an increase compared to the statistics from 2018 (2, 4). The absence of specific symptoms often leads to late diagnosis of laryngeal cancer, thereby resulting in delayed treatment. Local invasion, lymph node metastasis, distant metastasis, and pathological differentiation are the primary factors contributing to the unfavorable prognosis of laryngeal cancer. Thus, the exploration of novel biomarkers capable of predicting the prognosis of laryngeal cancer is crucial for early detection and intervention.

The development of laryngeal cancer is attributed to the activation of oncogenes and the inactivation of tumor suppressor genes, leading to dysregulated cell proliferation (3). Ki67 is a commonly utilized proliferation marker in laryngeal cancer. It is a nuclear protein associated with cell proliferation and is expressed during active phases of the cell cycle, except in the G0 phase, indicating cell proliferation (5). The proliferative index of Ki67 is closely linked to tumor differentiation, TNM staging, and prognosis. Consequently, it is widely employed to assess the proliferation of various malignant tumor cells (6).

p16 protein is a crucial tumor suppressor gene that belongs to the INK4 class of cell cycle inhibitors. It is located on chromosome 9p21 and acts as a cell cycle-dependent kinase inhibitor during the G1-S phase transition. By inhibiting cyclin-dependent protein kinases, p16 prevents entry into the S phase of the cell cycle, leading to the dephosphorylation of the Rb protein. Consequently, p16 negatively regulates cell proliferation and division, and its loss of function can contribute to tumor transformation (6, 7). p16 plays a significant role in the development and progression of various tumors, such as head and neck tumors, cervical cancer, esophageal cancer, liver cancer, and skin cancer (6–8). The loss of p16 function is frequently observed in these tumors, often resulting from gene deletion, mutation, or epigenetic silencing.

Previous clinical evidence has shown that the age of tumor onset, primary site, histological differentiation type, and cervical lymph node metastasis are associated with prognosis. However, these factors alone are not sufficient to evaluate the malignancy of tumors. Therefore, there is an urgent need to identify biomarkers that can predict the prognosis of laryngeal cancer patients. The aim of this study is to explore the expression of p16 protein and Ki67 proliferation marker in laryngeal squamous cell carcinoma and investigate their potential roles as prognostic biomarkers in laryngeal cancer.

## 2 Materials and methods

### 2.1 Study population

**Figure 1:**
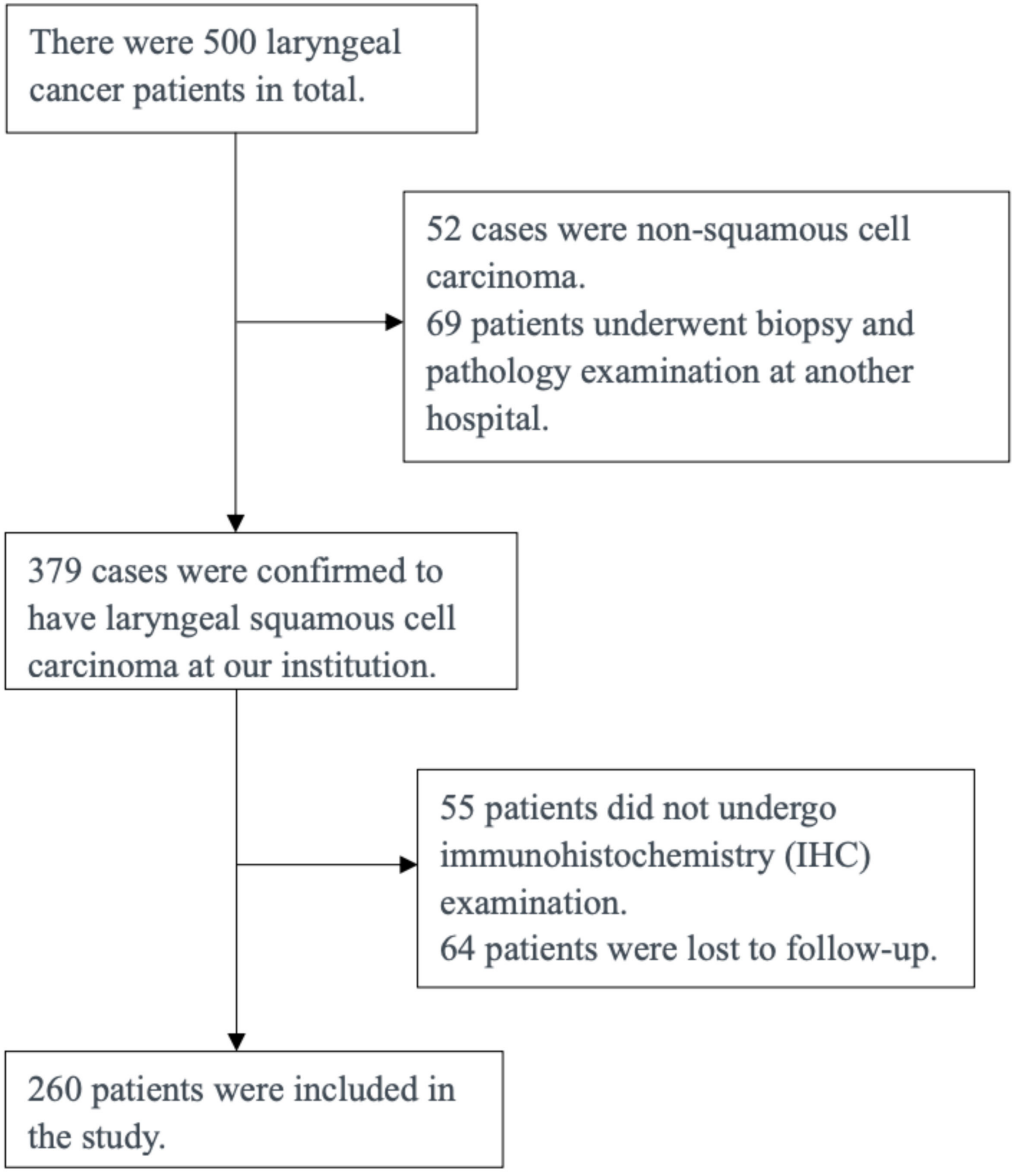
Incorporate the patient flowchart.

The following is a single-center investigation encompassing a total of 260 patients diagnosed with primary laryngeal cancer, who underwent treatment at the Department of Otolaryngology-Head and Neck Surgery, the First Affiliated Hospital of Xiamen University, spanning from 2012 to 2022 (**Figure 1**). Inclusion Criteria:(1) The biopsy and pathology examinations were completed at our institution. (2) Pathological type: squamous cell carcinoma;(3) Consent to undergo comprehensive immunohistochemical testing;(4) Clear clinical staging. Among the patients, 260 were male, with ages ranging from 40 to 95 years and a mean age of 59.4 years. The clinical data collected for the patients included age, gender, smoking and alcohol history, TNM staging, and histological classification. The distribution of laryngeal cancer types among the patients was as follows: 42 (16.1%) had supraglottic cancer, 160 (61.5%) had glottic cancer, 18 (6.9%) had subglottic cancer, and 40 (15.3%) had transglottic cancer. Histological grading revealed 77 cases of well-differentiated squamous cell carcinoma, 157 cases of moderately differentiated squamous cell carcinoma, and 26 cases of poorly differentiated squamous cell carcinoma. In terms of T staging, 144 patients were classified as T1-T2 and 116 patients as T3-T4. Additionally, the clinical TNM staging indicated that 137 cases were stage I-II and 123 cases were stage III-IV. Informed consent was obtained from all patients prior to enrollment, and the study was approved by the Ethics Committee of the First Affiliated Hospital of Xiamen University.

### 2.2 Methods

Immunohistochemical detection of p16 and Ki67 involves several steps, including specimen collection, preparation, staining, and result analysis.

1. Specimen collection: Representative cell samples are selected from postoperative pathological tissues.
2. Preparation: The collected cell samples are made into thin cell smears. After drying, they are fixed with a fixative to protect the cell structure and prevent cell fragmentation and protein degradation.
3. Staining: Immunohistochemical methods are used to detect the expression of p16 and Ki67 proteins. First, anti-p16 (Manufacturer : Roche Dlagnostlcs GmbH. Isotype: IgG.Monoclonal) and anti-Ki67 antibodies (Fuzhou Mai Xin Biotechnology Development Co., Ltd. Isotype: IgG.Monoclonal) are added to the cell smears. After a specific incubation period, corresponding secondary antibodies and substrates are added to achieve staining.
4. Result analysis: The cell smears are observed under a microscope to evaluate the expression of p16 and Ki67 proteins.

### 2.3 Statistical methods

Categorical and continuous variables are presented as counts and the mean (standard error), respectively. The Chi-square test was employed to assess variations in age, gender, alcohol and smoking history, tumor staging, tumor histological classification, and tumor type among the different Ki67 proliferation marker/p16 protein expression groups. Additionally, the Kaplan-Meier method was utilized to estimate median survival time, and the log-rank test was applied to statistically compare survival curves. Furthermore, a COX regression model was developed to evaluate the influence of Ki67 expression on the prognosis and survival risk of laryngeal cancer. A significance level of *P*<0.05 was set for all analyses.

## 3 Results

The data presented in **Table 1** indicates a gradual decrease in the survival time of patients with laryngeal cancer as the Ki67 index level increases, with significant differences observed in pathological classification and tumor staging (*P*<0.05). Moreover, there was a lower incidence of supraglottic cancer and a higher prevalence of transglottic cancer. Conversely, no significant differences in population characteristics were observed in the p16 (-/+) group (*P*>0.05).

**Table 1:** Basic demographic characteristics of laryngeal cancer patients with different P16 and Ki67 expression levels.

Features	Cases, n	Expression of p16, n		*P*-value	Expression of Ki67, n			*P*-value
		−	+		Ki67(0~35%)	Ki67(36%~70%)	Ki67(71%~100%)	
N	260	224	36		71	149	40	
Age	–	62.6 ± 9.7	63.6 ± 10.7	0.585	62.873 ± 9.096	63.503± 10.175	59.900 ± 9.535	0.120
Smoking				0.618				
No	140	122	18		41	80	19	0.582
Yes	120	102	18		30	69	21	
Drinking				0.674				0.983
No	167	145	22		46	95	26	
Yes	93	79	14		25	54	14	
Pathological diagnostic typing				0.021				<0.001
Low-differentiated squamous cell carcinoma	26	23	3		3	18	5	
Medium-differentiated squamous cell carcinoma	157	128	29		32	93	32	
Highly-differentiated squamous cell carcinoma	77	73	4		36	38	3	
Site of cancer				0.44				0.028
Acoustic portal	160	141	19		54	88	18	
Suprasegmental type	42	33	9		5	25	12	
Subglottal type	18	16	2		4	11	3	
Transonic portal	40	34	6		8	25	7	
Cancer Staging				0.53				<0.001
I	88	78	10		39	45	4	
II	49	44	5		14	31	4	
III	39	33	6		9	21	9	
IV	84	69	15		9	52	23	
Surgical history				0.571				0.307
No	12	11	1		1	9	2	
Yes	248	213	35		70	140	38	
Tertile of surivial time				0.124				<0.001
Q1(0.08-1.54)	80	65	15		14	51	15	
Q2(1.58-3.42)	93	79	14		17	61	15	
Q3(3.46-10.54)	87	80	7		40	37	10	

**Figure 2:**
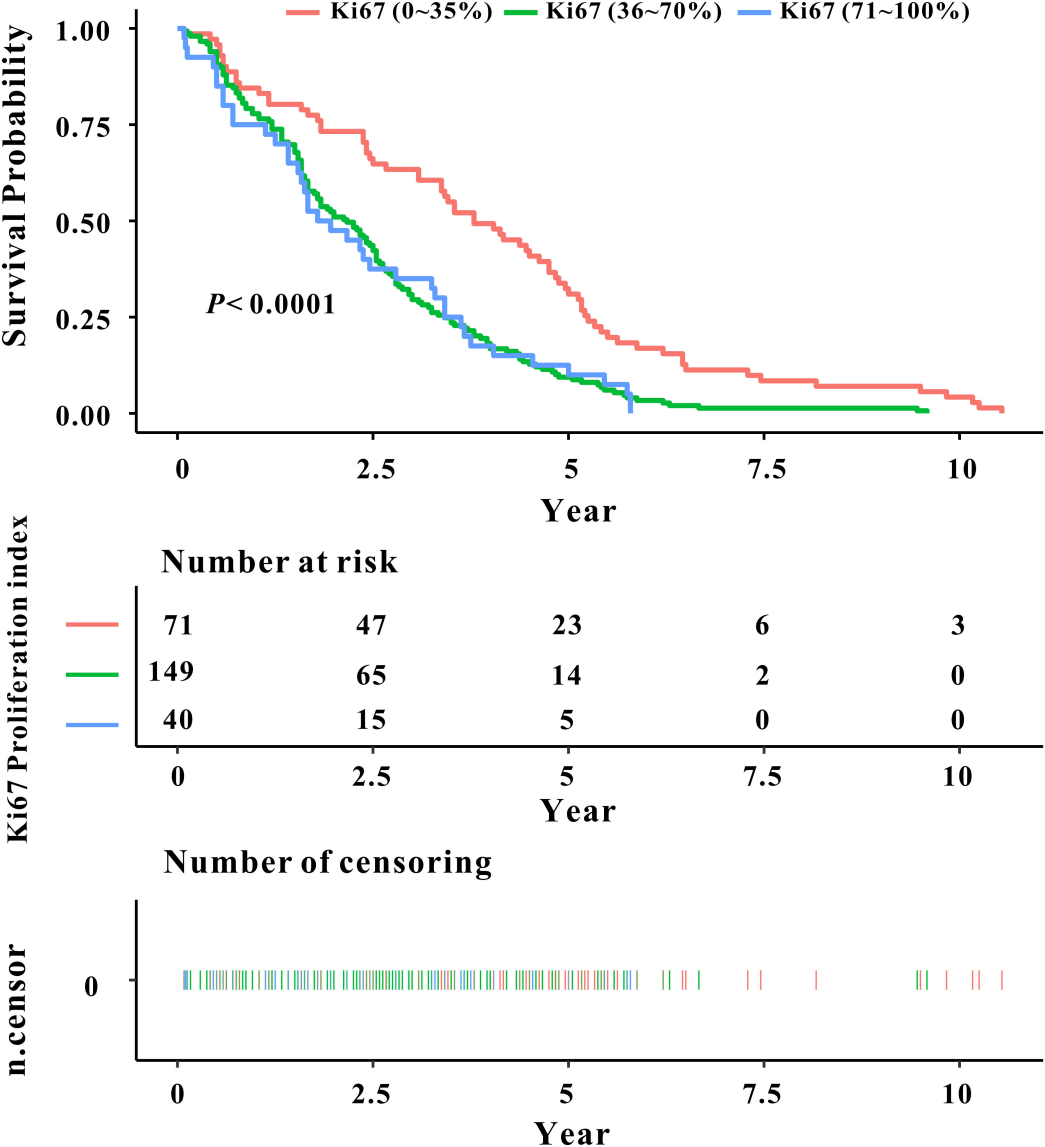
Kaplan-Meier curve depicting the relationship between Ki67 classification and prognosis survival rate in laryngeal cancer.

**Figure 3:**
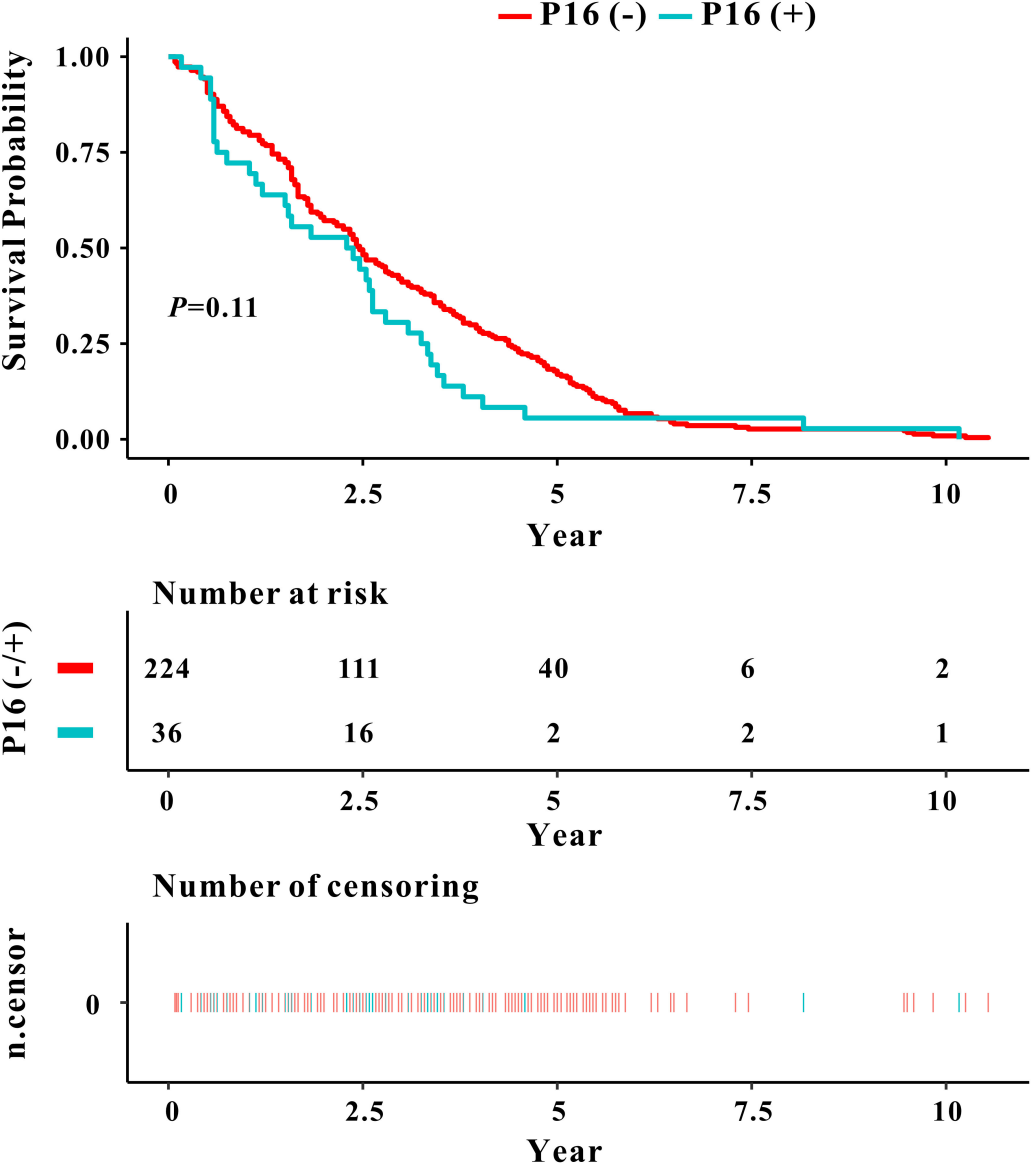
Kaplan-Meier curve depicting the relationship between P16 classification and prognosis survival rate in laryngeal cancer.

The analysis of patient survival time through Kaplan-Meier (KM) survival curve, as depicted in **Figure 2**, illustrated a decrease in the survival rate of laryngeal cancer patients with an escalation in the Ki67 index level (*P*<0.0001). Median survival times for Ki67 (0-35%), Ki67 (36-70%), and Ki67 (70-100%) were approximately 3.54 years, 2.10 years, and 1.92 years, respectively. Additionally, **Figure 3** demonstrated no statistically significant difference in survival rates between the p16 (-) and p16 (+) laryngeal cancer groups.

The study's findings demonstrated statistically significant differences in survival time among laryngeal cancer patients with varying Ki67 index levels. Consequently, a COX regression model was employed to assess the impact of Ki67 expression on the prognosis and survival of laryngeal cancer. In **Table 2**, the regression model's outcomes indicated that, independent of other variable factors, higher Ki67 index levels were associated with an increased risk of death from laryngeal cancer (*P*<0.001). Following adjustments for variables such as age, smoking, drinking, pathological classification, surgery, recurrence, and metastasis, the risk of death in the Ki67 (70-100%) laryngeal cancer group was determined to be 2.0504 times (1.2997, 3.2345) higher than that in the Ki67 (0-35%) index level group (*P*=0.0020).

**Table 2:** COX regression analysis of Ki67 index level with the risk of poor prognosis and death in laryngeal cancer.

Exposure	Model 1^a^	Model 2^b^	Model 3^c^
ki67 Proliferation index (continuous)	1.5153(1.2640,1.8156)	1.5593(1.2872,1.8888)	1.4629(1.1765,1.8189)
ki67 Proliferation index			
Ki67(0~35%)	1	1	1
Ki67(36%~70%)	2.0398(1.5151,2.7640) 0.0001	2.0319(1.5059,2.7417) 0.0001	1.7412(1.2535,2.4187 0.0009
Ki67(71%~100%)	2.1300(1.4304,3.1879) 0.0002	2.2081(1.4508,3.3606) 0.0002	2.0504(1.2997,3.2345) 0.0020
P-trend	<0.0001	<0.0001	0.0006

Model 1: Unadjusted variable.

Model 2: Age, smoking, alcohol intake.

Model 3: Age, smoking, alcohol intake, histological subtype, surgical status, recurrence status, metastasis status.

## 4 Discussion

Ki67 is widely recognized as a critical marker of cell proliferation, exerting a pivotal role in DNA synthesis and mitosis. Elevated Ki67 expression in various cancer types often correlates closely with increased tumor cell proliferation and invasion. In the study conducted by Bai et al., a noticeable increase in Ki67 expression was observed in laryngeal squamous cell carcinoma tissues compared to adjacent non-tumor tissues (9). Furthermore, high Ki67 expression was significantly linked to cervical lymph node metastasis, indicating its potential utility as a significant prognostic indicator for laryngeal squamous cell carcinoma. Several research studies have also established an association between Ki67 expression and tumor staging in laryngeal cancer, underscoring its relevance in the evaluation of prognosis (7, 10, 11). Thus, Ki67 stands as a valuable marker, offering insights into tumor progression and invasiveness within the context of laryngeal cancer.

The current investigation primarily examined the expression of Ki67 in laryngeal cancer and its association with tumor staging, survival duration, and mortality risk. Our findings indicate a progressive decrease in the survival time of laryngeal cancer patients as the Ki67 index level escalates, accompanied by significant disparities in pathological classification and tumor staging (*P*<0.05). Furthermore, heightened Ki67 expression was positively linked to an elevated risk of mortality in laryngeal cancer patients. These outcomes underscore the pivotal role of Ki67 in the initiation, progression, and prognosis assessment of laryngeal cancer. Elevated Ki67 expression may signify heightened malignancy, invasiveness, and metastatic potential in laryngeal cancer. Consequently, Ki67 stands to offer valuable potential as a prognostic indicator in laryngeal cancer, thus furnishing valuable insights for clinical diagnosis and treatment. The assessment of Ki67 aids pathologists in more precise evaluation of tumor proliferation, providing essential guidance for staging and classification of the disease. Moreover, the expression level of Ki67 is closely associated with survival rates in patients with laryngeal cancer, where higher Ki67 expression typically correlates with poorer prognosis. This characteristic can serve as a reference indicator for predicting patient response to treatment and survival outcomes in clinical settings.

The p16 protein, encoded by the CDKN2A gene, functions as a tumor suppressor by inhibiting cyclin-dependent kinase 4, thereby preventing cell cycle entry into the S phase. This action helps to maintain the low phosphorylation state of Rb and prevents its dissociation from E2F transcription factors, playing a vital role in cell growth regulation. Nevertheless, there remains a lack of consensus regarding the application of p16 in laryngeal cancer (12). Some studies have suggested that patients with p16-positive tumors exhibit a more favorable prognosis, less invasive cancer behavior, and reduced risks of lymph node and distant metastasis (13). Conversely, other research has yielded no significant difference in outcomes between p16-positive and p16-negative patients (14). These discrepancies may be attributed to limitations in research methodologies, small sample sizes, and the heterogeneity of patient populations.

In this investigation, we conducted an analysis of p16 expression in laryngeal cancer patients and assessed its correlation with clinical and pathological characteristics. Our findings revealed that p16 positivity exhibited no statistically significant correlation with average age, smoking, drinking, tumor staging, lymph node metastasis, or prognosis survival rate in our study cohort. While the expression of p16 in laryngeal cancer did not demonstrate a significant association with tumor staging or survival time, it remains possible that p16 may still play a role in the pathogenesis and prognosis assessment of laryngeal cancer. Subsequent studies are warranted to provide further elucidation on the role of p16 in laryngeal cancer, potentially offering novel insights for clinical diagnostic and treatment strategies.

While this study boasts strengths in terms of sample size and follow-up duration, it does come with certain limitations. Firstly, being a single-center, retrospective study, there is potential for selection bias and information bias. Secondly, the study fails to adjust for other confounding factors, such as the patient's nutritional status and mental health, which could also influence outcomes. We intend to embark upon a larger-scale multicenter clinical investigation to significantly augment the robustness and generalizability of our findings. The findings of this study affirm a substantial correlation between heightened Ki67 expression and the growth and invasive capabilities of laryngeal cancer. As a marker of cell proliferation, Ki67 holds promise as a significant molecular target for the diagnosis, treatment, and prognosis evaluation of laryngeal cancer. Nevertheless, additional research is imperative to fully elucidate the specific mechanisms through which Ki67 operates in laryngeal cancer and its interrelation with other molecular markers.

## 5 Conclusion

In summary, the findings of this study indicate a strong correlation between the Ki67 index level and the prognosis, survival time, and risk of mortality in laryngeal cancer, thereby offering significant predictive value. Conversely, the prognostic value of p16 levels in laryngeal cancer appears to be limited. These insights provide substantial reference points for the prognosis assessment and therapeutic decision-making processes in patients with laryngeal cancer.

## Data Availability

Requests to access these datasets should be directed to chenlinteng888@vip.qq.com.
